# A QM/MM Study of Nitrite Binding Modes in a Three-Domain Heme-Cu Nitrite Reductase

**DOI:** 10.3390/molecules23112997

**Published:** 2018-11-16

**Authors:** Kakali Sen, Michael A. Hough, Richard W. Strange, Chin W. Yong, Thomas W. Keal

**Affiliations:** 1 School of Biological Sciences, University of Essex, Wivenhoe Park, Colchester, Essex CO4 3SQ, UK; kakali.sen@stfc.ac.uk (K.S.); mahough@essex.ac.uk (M.A.H.); rstrange@essex.ac.uk (R.W.S.); 2 Scientific Computing Department, STFC Daresbury Laboratory, Warrington, Cheshire WA4 4AD, UK; chin.yong@stfc.ac.uk

**Keywords:** nitrite reductases, three-domain CuNiRs, *Rp*NiR, nitrite binding, QM/MM methods

## Abstract

Copper-containing nitrite reductases (CuNiRs) play a key role in the global nitrogen cycle by reducing nitrite (NO_2_^−^) to nitric oxide, a reaction that involves one electron and two protons. In typical two-domain CuNiRs, the electron is acquired from an external electron-donating partner. The recently characterised *Rastonia picketti* (*Rp*NiR) system is a three-domain CuNiR, where the cupredoxin domain is tethered to a heme *c* domain that can function as the electron donor. The nitrite reduction starts with the binding of NO_2_^−^ to the T2Cu centre, but very little is known about how NO_2_^−^ binds to native *Rp*NiR. A recent crystallographic study of an *Rp*NiR mutant suggests that NO_2_^−^ may bind via nitrogen rather than through the bidentate oxygen mode typically observed in two-domain CuNiRs. In this work we have used combined quantum mechanical/molecular mechanical (QM/MM) methods to model the binding mode of NO_2_^−^ with native *Rp*NiR in order to determine whether the N-bound or O-bound orientation is preferred. Our results indicate that binding via nitrogen or oxygen is possible for the oxidised Cu(II) state of the T2Cu centre, but in the reduced Cu(I) state the N-binding mode is energetically preferred.

## 1. Introduction

Microbial copper-containing nitrite reductases (CuNiRs) are enzymes that catalyse the reduction of NO_2_^−^ to NO, a key denitrification step in the global nitrogen cycle [1]. The most studied and well-characterized are the two-domain CuNiRs, which are homotrimeric proteins with each monomer consisting of two cupredoxin-like domains containing an electron-transfer type 1 Cu (T1Cu) site and a catalytic type 2 Cu (T2Cu) site [2,3]. The T1Cu is typically coordinated with Cys-Met-His_2_ and the T2Cu is coordinated with His_3_-H_2_O in the resting phase. The two sites are separated by approx. 12.5 Å and are connected by a Cys-His bridge, with Cys coordination to the T1Cu and His to the T2Cu. Catalytic reduction occurs at the T2Cu site after binding of NO_2_^−^ via replacement of the bound water and involves a proton-coupled electron transfer reaction: NO_2_^−^ + 2H^+^ + e^−^ → NO + H_2_O. The electron is internally transferred from the T1Cu site to the T2Cu site via the Cys-His bridge and the T1Cu in turn receives an electron from external redox proteins such as azurins and pseudoazurins or c-type cytochromes [2].

**Figure 1:**
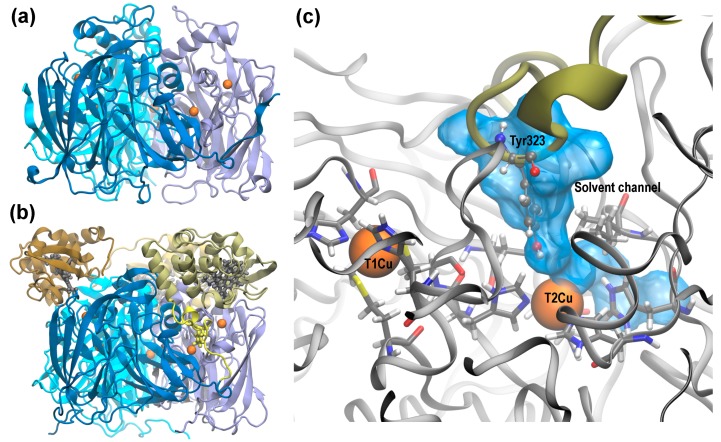
Structural similarities in two domain *Achromobacter cycloclastes* CuNiR (*Ac*NiR) and three domain *Rp*NiR: (**a**) Structure of *Ac*NiR, PDB ID: 2BW4. (**b**) Structure of *Rp*NiR, PDB ID: 3ZIY. The T1 and T2 Cu ions are represented as orange spheres. The heme *c* units of the cytochrome domain in *Rp*NiR are shown in grey. The linker chain that tethers the cupredoxin domain and the cytochrome domain is shown in yellow along with Tyr323 which is strategically positioned in the solvent/ligand accessible channel. (**c**) Structures of *Rp*NiR and *Ac*NiR are overlapped and zoomed in at the T2Cu site. The backbone and active site residues from *Ac*NiR are shown. The critical position of Tyr323 in *Rp*NiR blocks the otherwise solvent/ligand accessible channel (shown in blue) in the two-domain CuNiRs. The heme *c* domain linker from *Rp*NiR that strategically positions Tyr323 is shown in green.

In recent years, two new sub-classes of CuNiRs have been identified that, in addition to the core two-domain structure, have a cupredoxin or heme *c* domain fused to the N- or C-terminus respectively [4,5,6,7]. Genomic analysis revealed a wide distribution of this novel type of CuNiR in several organisms [8]. The first three-domain CuNiR with a cytochrome *c* domain fused to the core two-domain cupredoxin to be biochemically characterized was isolated from *Rastonia picketti* (*Rp*NiR) with its structure determined to 1.01 Å resolution [7,9]. The structure reveals it to be trimeric with high structural similarity to the cupredoxin domain in two-domain CuNiRs such as *Ac*NiR (Figure 1a,b). The heme *c* domain of one monomer is in close proximity to the T1Cu site from another monomer, with the heme Fe ion and T1Cu unit within 10.4 Å of each other, an acceptable electron transfer distance (Figure 2 in [9]), and hence the heme *c* is capable of supplying electrons to T1Cu during turnover.

There is a striking similarity between the core cupredoxin domain of *Rp*NiR and the two-domain *Ac*NiR (Figure 1a,b). However, the additional heme domain necessitates certain rearrangements in the core structure. In two-domain CuNiRs, X-ray studies revealed the presence of two solvent access channels with the potential to deliver protons from the bulk solvent to the T2Cu site during the reduction of NO_2_^−^, one of which also functions as the substrate access channel [10,11]. Structural, spectroscopic and computational studies have revealed the importance of the substrate access channel in controlling the hydration of the active site [12,13,14], the orientation of the nitrite and selectivity for small ligands [15,16], and determining the rate of turnover [12]. In *Rp*NiR, the linker that tethers the heme *c* domain to the cupredoxin domain is poised in such a way that the sidechain of one of its residues, Tyr323, resides close to the T2Cu site, thereby blocking this substrate channel (Figure 1c). The position of the Tyr323 is critical and it is hypothesized to have mechanistic implications. Unusually, the three-domain *Rp*NiR has a remarkably low affinity for binding NO_2_^−^ in the oxidized T2Cu state [7].

Another striking similarity among all CuNiRs is the presence of conserved Asp and His residues in the T2Cu active site, which are believed to participate in the proton transfer that is necessary for reduction [16,17,18,19]. In *Rp*NiR the corresponding Asp and His are Asp97 and His240, respectively. A mutant of *Rp*NiR in which the catalytically important Asp97 was mutated to Asn (D97N) [20] revealed that binding of NO_2_^−^ was possible after soaking the NO-bound mutant with sodium nitrite, where NO is replaced by NO_2_^−^. The D97N NO-bound structure revealed a displacement of Tyr323 from the substrate access channel, associated with adjustment of the linker loop (Ser315–Ser321), thereby opening up the substrate access channel and allowing NO_2_^−^ to bind. In this mutant, NO_2_^−^ is bound via the nitrogen atom rather than the bidentate η^2−^O,O (‘top-hat’) mode observed in the crystal structures of several two-domain CuNiRs [10,21,22,23].

The focus of this work is on modelling the NO_2_^−^ binding mode at the T2Cu site in native *Rp*NiR, following ligand entry into the active site region. The *K*_m_ of *Rp*NiR for nitrite is similar to that of other two-domain CuNiRs in which the nitrite ion is observed in crystal structures to bind in a bidentate ‘top-hat’ orientation. The recent development of Multiple Structures from One Crystal (MSOX) methodology has led to the creation of structural movies of NO_2_^−^ reduction at the T2Cu site in the two-domain *Ac*NiR [21]. One of the features was the change of NO_2_^−^ binding mode from top hat to a ‘side-on’ orientation where both the oxygen and nitrogen atoms are equidistant from T2Cu (Figure 2 in [21]), which is thought to occur as a result of reduction of the T2Cu. In addition, the mode of nitrite binding is important for mechanistic understanding of the reduction of nitrite in two-domain CuNiRs, which may proceed according to either an ordered [24,25,26] or random-sequential mechanism [27,28] and there is similar speculation about the mechanism in three-domain *Rp*NiR [7,20]. As binding via either O or N is possible for the nitrite ligand, it is essential to identify the initial binding mode of nitrite in the active site to elucidate the mechanism for *Rp*NiR. Until now no direct structural information of the binding mode of NO_2_^−^ in native *Rp*NiR is available, although binding via nitrogen was observed for the D97N mutant [20]. We have therefore used combined quantum mechanical and molecular mechanical (QM/MM) methods to determine whether binding of NO_2_^−^ occurs via the N- or O-atom in native *Rp*NiR and have compared the calculated structures to the available experimental data for the D97N mutant as well as the available structural data on two-domain CuNiRs. 

## 2. Results

### 2.1. Molecular Dynamics of Native and D97N RpNiR in the Resting State 

Initial classical MD simulations were carried out to investigate the positioning of the crucial active site residues such as Tyr323 and provide snapshots for subsequent QM/MM calculations. Analogously to other CuNiRs, the conserved active site Asp residue, Asp97 in *Rp*NiR is likely a key player for the proton transfer required during catalytic reduction at the T2Cu. We therefore ran simulations of the resting states of three *Rp*NiR trimer systems: the two protonation states of Asp97 (which we label “D97” and “D97p” for the unprotonated and protonated states respectively) and an in silico mutated structure “D97N” where Asp97 is replaced with Asn. Conformational variations within the T2Cu active sites of the three independent subunits (chains A, B and C) of the trimer were analysed over the unrestrained last 75 ns of a total of 80 ns of MD trajectories for each of these three systems (see the methodology section for further details).

**Figure 2:**
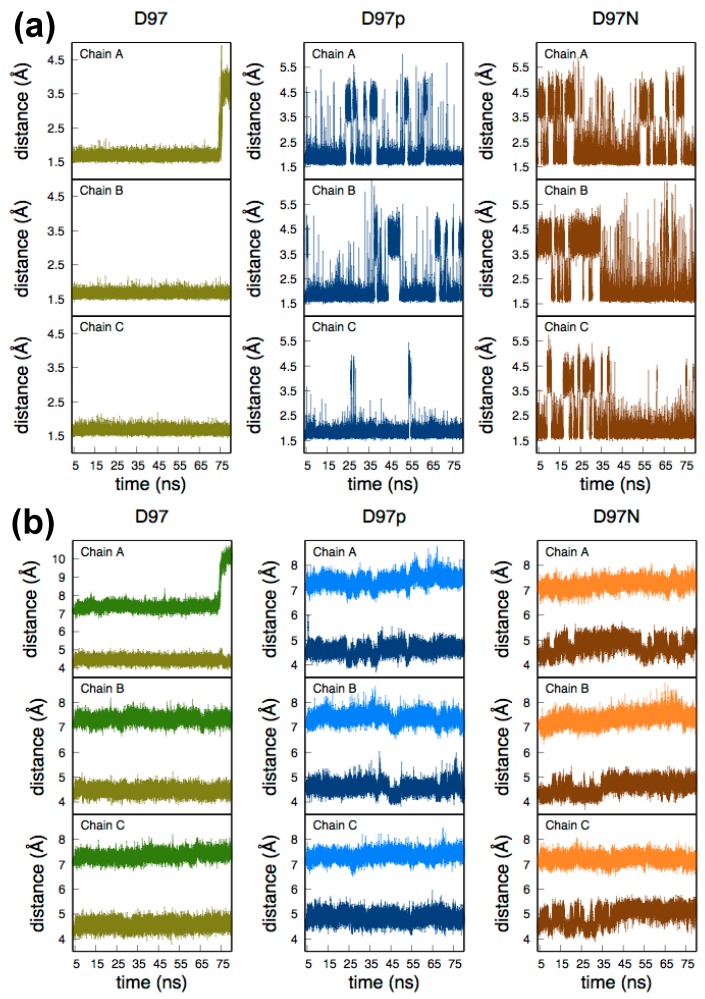
Relevant distances from 75 ns of MD trajectories for the three independent subunits (chains A, B and C) of *Rp*NiR, simulating deprotonated (D97) and protonated (D97p) active site Asp97 and the Asn97 mutant (D97N). (**a**) The distance between Tyr323-phenolic H to the nearest O of Asp97 (for D97 and D97p) and to O/N- of Asn97 (for D97N). (**b**) The distance between (i) the T2Cu ion and the centre of mass of the side chain of Asp97 (olive in left panel and dark blue in middle panel) and that of Asn97 (brown in right panel); (ii) the T2Cu ion and the centre of mass of the side chain of Tyr323 (green in left panel, light blue in middle panel and orange in right panel).

As the Tyr323 residue sits in the substrate access channel it interacts with the hydration network connecting Asp97, T2Cu and His240. Figure 2a depicts the dynamic hydrogen bond distance between the phenolic H atom of Tyr323 and the nearest O atom of Asp97/nearest N- or O-atom of Asn97. In the case of D97, a strong hydrogen bond is present between the negatively charged carboxylic acid group of Asp97 and Tyr323. Protonation of Asp97 weakens this interaction and as a result a transient break in this interaction is frequently observed in the D97p system. A similar situation is observed for the D97N mutant with the transient breaks becoming more frequent. Interestingly, there is a complete breaking of the TyrO-H···O-Asp97 interaction at ~69 ns in one subunit of the D97 system (chain A in Figure 2a). This is due to movement of the Tyr323 away from the T2Cu pocket (*vide infra*). To understand the relative position of these two amino acids with respect to the T2Cu ion, the time evolution of the distances between the T2Cu and the centre of mass (COM) of the side chains of Tyr323 and Asp97 during MD were measured and are plotted in Figure 2b, while the averaged values over the entire trajectory for each subunit are given in Table 1. The average distances between Asp97 and T2Cu are similar to those in the crystal structure. The average distance of Asn97-T2Cu for the mutant D97N simulations is larger than that measured in the recent D97N crystal structure [20] (4.30 Å). The other distance, T2Cu and COM of the Tyr323 side chain, in the native crystal geometry is 7.16 Å. The average values for the T2Cu-Tyr323 distance in D97, D97p and D97N systems also agree well with the crystal structure (Table 1).

The exception, as noted above, is in one subunit of D97 (chain A in Figure 2b and Table 1) where the Tyr323 becomes displaced from the T2Cu site by 2.5–3.0 Å after ~69 ns. Since the relative positions of Asp97 and T2Cu are unchanged in the same MD timeframe, this implies that it is the flexibility of the linker chain that enables displacement of the Tyr323. Although the orientations of the Tyr323 sidechain in the two cases are different, the displacement seen by MD corroborates the flexibility observed in the D97N crystal structure (Figure S1), where the sidechain is rotated ~90° away from its position in the native crystal structure (Figure 2a in [20]).

In the D97p and the D97N systems (Figure 2a, middle and right panels) the local H-bond network between Tyr323 and Asp/Asn97 is frequently disrupted. However, within the timescale of measurement Tyr323 remains ‘locked’ in its position in the substrate channel and so would sterically hinder the entry of NO_2_^−^. In the D97 system Tyr323 is held strongly in H-bond interaction by the negatively charged Asp97 but in the case of chain A the Tyr323 nevertheless breaks away towards the end of the simulation (Figure 2b, left panel), becoming displaced in the channel by 2.5-3.0 Å from its original position relative to the T2Cu (Figure S1). The distance of Tyr323 from T2Cu in chain A (Table 1) at ~69 ns is similar to that found by Dong et al. in the crystal structure of NO- and nitrite-bound D97N *Rp*NiR [20].

### 2.2. QM/MM Optimization of NO_2_^−^ Bound Structures

Snapshots of structures from the MD simulations were taken as the basis for studying the binding mode of nitrite to the T2Cu site. The snapshots were selected to be representative of the conformational variations observed in the MD trajectories described in the previous section, taking them from 25 ns onwards to ensure that the systems were completely equilibrated after removal of backbone constraints. The nitrite ion was added to the systems as described in the methodology section. QM/MM optimizations were carried out for both the Cu(II) and Cu(I) states of the T2Cu ion, starting from both ‘top-hat’ and ‘N-bound’ orientations of NO_2_^−^. For each optimisation, the geometry and relative energy of the relaxed structure were assessed.

We categorised the relaxed geometries of the T2Cu site in two ways. First, by the overall geometry of the T2Cu-nitrite complex, which can be either tetragonal or trigonal. This was determined by measuring the twisting angle φ between two planes: one defined by N(NO_2_^−^)-T2Cu-N(His1) and the other defined by N(His2)-T2Cu-N(His3), following the procedure used by Ryde et al. for blue and green Cu proteins [29,30]. His1 is the axial ligand, defined as the ligand with the largest N(NO_2_^−^)-T2Cu-N(His) angle, with His2 and His3 the remaining equatorial ligands. For a tetragonal structure φ will be approximately 0° or 180°, while for trigonal structures φ will be approximately 90°.

**Figure 3:**
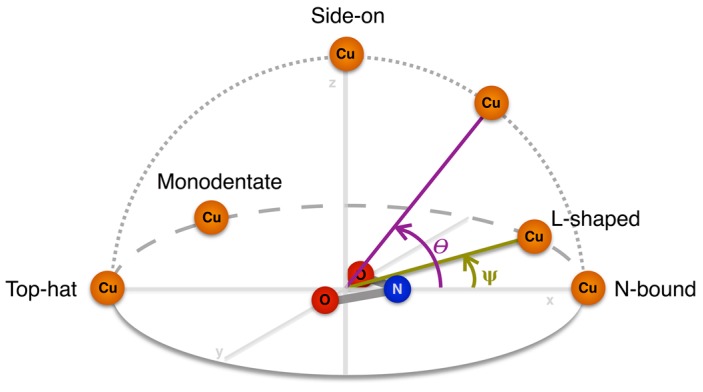
Schematic representation of the geometric angles θ and ψ that define the orientation of NO_2_^−^ bound to T2Cu.

The second assessment was of the orientation of the NO_2_^−^ ion bound to the T2Cu centre. The nitrite orientation can be classified as symmetrical N-bound, L-shaped, bidentate top-hat, monodentate top-hat, or side-on. To assign these labels we define a coordinate system in which the centre of mass of NO_2_^−^ is placed at the origin with the nitrogen atom aligned along the positive X-axis, and the oxygen atoms lie on the XY plane, as shown in Figure 3. Two angular measurements describing the position of Cu in relation to this coordinate system can then be used to classify the different orientations. We define an altitudinal angle θ as the angle between the projection of Cu onto the XZ plane and the X-axis. This angle can be thought of as the ‘pitch’ of the NO_2_^−^ ion and describes a change in its orientation from N-bound (0°) to side-on (90°) to top-hat (180°). We also define an azimuthal angle ψ as the angle between the projection of Cu onto the XY plane and the X-axis. This angle can be thought of as the ‘yaw’ of the NO_2_^−^ ion which circles from N-bound (0°) to l-shaped (~30°) to perpendicular (90°, not observed in practice) to monodentate top-hat (~150°) to bidentate top-hat (180°). The thresholds we use for labelling the nitrite ion orientation in each structure are shown in Table 2.

The energies of the relaxed conformers were assessed to determine whether there was any preference for one binding mode over another. Absolute energies between the conformers cannot be compared since the total number of atoms of the conformers is not conserved as they are taken from random time steps and a shell of solvent is used. Instead a relative energy was calculated from the difference in energy between relaxed top-hat and N-bound structures (ΔE = E_top-hat_ – E_N-bound_) such that a positive ΔE indicates that N-bound is favoured while a negative value indicates top-hat is favoured.

#### 2.2.1. D97 *Rp*NiR

Figure 2 indicates that within the timescale of the MD simulation the majority of the Tyr323 conformations are hydrogen bonded to Asp97 and positioned well into the substrate channel. However, a fraction of the conformations involves a disruption of the Tyr323-Asp97 hydrogen bond and a major displacement of Tyr323 from its position in the substrate channel. We therefore selected two snapshots to represent these possibilities. The first of these was taken at 72.53 ns where Tyr323 maintained an H-bond interaction with Asp97 in all three subunits. It represents the major conformational orientation observed from MD in this system (Figure 2a, left panel). The second snapshot was taken at 79.60 ns where the Tyr323 is displaced from the active site (Figure 2a,b, left panel) breaking its H-bond interaction with Asp97 in chain A, but in chains B and C it resides in the active site and forms an H-bond interaction with Asp97. As detailed in the Methods section optimizations were carried out separately for each monomeric unit, resulting in six independent starting structures. Relative energies and geometric descriptions of these are presented in Table S1.

1) Geometries of optimized Cu(II) and Cu(I) states starting from top-hat and N-bound orientations of NO_2_^−^

In the case of Cu(II) starting from a top-hat orientation, all the relaxed structures retain the top-hat orientation, with both θ and ψ within 170–180°. The nitrite oxygen atoms are roughly equidistant from Cu, with distances differing by ~0.3 Å (Table S1). When reduced to Cu(I), the relaxed coordination changes to monodentate top-hat orientation with asymmetric Cu-O distances. θ lies within the range from 160° to 180°, with the majority of structures in the 170–180° range, while ψ is around 145–165°, a signature of monodentate NO_2_^−^ in top-hat orientation. Here Cu is bound to O, hence N is necessarily displaced, resulting in the azimuthal angle of ~150°. In one conformation, θ is 159.1°, at the lower limit of the observed range.

Starting from N-bound orientations, there is more variation in the relaxed structures. All the final structures represent N-bound geometry with θ < 10°. However, both symmetric (ψ < 10°) and l-shaped (ψ > 10°) orientations are observed. This is also reflected in the two Cu-O distances: for l-shaped N-bound structures, the difference between the two Cu-O distances are >0.3 Å. On reduction to Cu(I) we observe θ < 10° as in Cu(II), but the N-bound structures are more symmetrical in nature, where ψ < 3° and in many cases close to 0°, with the oxygen atoms therefore equidistant from Cu. The range of θ and ψ observed for this system across all the 6 structures are given in Table 3. The angle φ is in the range of 80–90° and six angles around Cu are in the range of 90–135°, but in some cases, one of the N(NO_2_^−^)-T2Cu-N(His) angles is larger and fell in the range of 140–162° (Table S1). Overall the structures can be classified as tetrahedrally distorted trigonal geometries. 

2) Energetics of Cu(II) and Cu(I) states

In the case of Cu(II), for snapshots where the Tyr323 is engaged in a H-bond interaction with Asp97, the structures are mostly close in energy, and in one structure the bidentate orientation is favoured over the l-shaped NO_2_^−^ orientation (Table S1). In the structure where Tyr323 is displaced from the T2Cu site, an extended H-bond network exists linking Tyr323 to Asp97 and the l-shaped N-bound structure has more favourable energy than the bidentate top-hat structure. The complex active site H-bond pattern as well as the interactions of the other active site residues is susceptible to the orientation of NO_2_^−^ at the T2Cu site and likely contributes to the difference in energy observed in these structures. In Table 4 the average energy difference ΔE between the top-hat and N-bound structures is presented. For Cu(II) the structures are effectively isoenergetic.

On reduction to Cu(I), the symmetrical N-bound orientation of NO_2_^−^ structures are energetically favoured over the corresponding monodentate top-hat structures. Unlike Cu(II), the D97 system indicates a clear trend in preference for a symmetrical N-bound orientation in the Cu(I) state (Table 4). Similar observations were obtained from previous calculations on other two-domain CuNiRs and model compounds representing the T2Cu site of CuNiRs [31,32].

#### 2.2.2. D97p *Rp*NiR

In contrast to the D97 system, protonation of Asp97 causes frequent transient disruption of the Tyr323-Asp97 H-bond (Figure 2a, middle panel), while in the timescale of the MD simulation no significant movement of Tyr323 from its position in the substrate channel is observed (Figure 2b, middle panel). Three representative snapshots were taken which differed by the number of monomeric units that featured the presence of a Tyr323-Asp97 H-bond: in all monomeric units (74.52 ns), in two of the three monomeric units (44.31 ns), and in one monomeric unit (48.40 ns) respectively. Optimization of these snapshots was carried out for all monomeric units, providing 9 independent conformations. Two structures in Cu(II) state resulted in orientations with very short Cu-O-Asp97 distance, which is not observed experimentally. These structures were discarded, resulting in 7 independent starting structures. Relative energies and geometric descriptions of all conformations are presented in Table S1.

1) Geometries of optimized Cu(II) and Cu(I) states starting from top-hat and N-bound orientations of NO_2_^−^

In the case of Cu(II) starting from top-hat orientation, all the relaxed structures optimized in a bidentate top-hat orientation, with both θ and ψ within the range 170–180°. The oxygens are approximately equidistant from Cu, with distances differing by ~0.3 Å. When reduced to Cu(I), the relaxed orientation changes to monodentate top-hat. In the majority of the structures θ is in the range of 170–180°. In two conformations, however, θ is 153.2° and 145.0°, respectively falling outside the range given in Table 2. Such cases fall in the borderline between the top-hat and side-on categories. The azimuthal angle ψ lies in the range 145–165°, the same as observed for the D97 system, which is a signature for monodentate top-hat orientation.

Similarly to the D97 system, the Cu(II) structures starting from an N-bound orientation show more structural variation than its Cu(II) top-hat counterpart. All the final structures feature an N-bound geometry with θ < 10°; however both symmetric (ψ < 10°) and l-shaped (ψ > 10°) orientations are observed. This is also reflected in the two Cu-O distances: for the l-shaped N-bound structures the difference between the two Cu-O distances are >0.3 Å (Table S1). On reduction to Cu(I), all N-bound structures have θ < 20° as shown in Table 2; with the majority within θ < 10°. The l-shaped orientation is not observed, rather all of them fall in the category of symmetrical N-bound orientation with equidistant O-atoms and ψ < 6°. In the majority of structures, the variation in ψ is even less; ψ < 3° (Table S1). The distribution of θ and ψ observed for this system are also provided in Table 3. The angle φ is in the range of 78–90° and six angles around Cu are in the range of 90–135° but in a few cases one of the N(NO_2_^−^)-T2Cu-N(His) angles is larger and fell in the range of 140–145° (Table S1). Like D97, the geometries can overall be classified as tetrahedrally distorted trigonal structures.

2) Energetics of Cu(II) and Cu(I) states

In the Cu(II) case the energy difference between individual top-hat and N-bound structures is usually less than 5 kcal/mol (see Table S1) which could arise from small changes in the active site H-bond network. There is no clear evidence of energetic differences arising from the orientation of the NO_2_^−^ ion. Unlike the D97 system, there is a small trend towards symmetrical N-bound structures over monodentate structures. Table 4 shows the average ΔE for both Cu(I) and Cu(II) states. For Cu(II) the structures are effectively isoenergetic with a small standard deviation, whereas for Cu(I) the average ΔE suggests that N-bound is favoured, but with a large standard deviation showing there is significant variations over the snapshots.

#### 2.2.3. D97N Mutant *Rp*NiR

As with the D97p system, a mutation of Asp97 to Asn weakens the hydrogen bond between Asn97 and Tyr323 causing frequent transient disruption of the H-bond interaction (Figure 2a, right panel). Another similarity with the D97p system is an absence of significant movement of Tyr323 from its initial position in the substrate channel (Figure 2b, right panel). Three representative snapshots were taken which differed according to the number of monomeric units for which the Tyr323-Asn97 H-bond was absent: in all monomeric units (25.54 ns), in two of the three monomeric units (27.86 ns) and in one monomeric unit (64.95 ns) respectively. An additional snapshot at 78.48 ns was added in the last category, to represent the situation where Tyr323 is H-bonded to O-Asn in one monomeric unit like the 64.95 ns snapshot and in the other is H-bonded via N-Asn. Optimization was carried out for each of the monomeric units providing 12 independent conformations, of which 3 conformations are not reported due to the presence of a short Cu-OAsn97 bond that is not observed in CuNiR crystal structures. This leaves a total of 9 conformations that are studied for this mutant system. Relative energies and geometric descriptions of all conformations are presented in Table S1.

1) Geometries of optimized Cu(II) and Cu(I) states starting from top-hat and N-bound orientations of NO_2_^−^

Optimization of top-hat Cu(II) structures resulted in bidentate top-hat orientation, with both θ and ψ within the range 170–180° as categorised in Table 2. The oxygen atoms are approximately equidistant from Cu, with distances differing by ~0.3 Å. When reduced to Cu(I), the relaxed coordination changes to a monodentate top-hat orientation. θ ranges from 160° to 180°, with the majority of structures within 170–180°. Unlike for D97 and D97p, in one conformation a side-on orientation of NO_2_^−^ is observed (θ and ψ ~88°). This is consistent with data for the two-domain CuNiR, whose T2Cu site lacks the linker residue [21]. Another conformation falls in the borderline of top-hat and side on with θ = 149.4°. In most of the structures the value of ψ is around 145–165° as observed for D97 and D97p systems and implies a monodentate top-hat orientation. There is another outlier, where ψ is 172.1° and is of bidentate nature. Again, as with the D97 and D97p systems more structural variations were observed starting from the N-bound NO_2_^−^ orientation. All the final Cu(II) structures feature an N-bound geometry with θ <10°; however symmetrical N-bound structures (ψ < 10°) are more abundant than the l-shaped N-bound (ψ > 10°) ones. In one N-bound case a penta-coordination around T2Cu is observed with symmetrical N-bound NO_2_^−^ and a water. On reduction to Cu(I), the structures relaxed to symmetrical N-bound orientation with θ < 10°, and in the majority of structures ψ < 3°. For two structures ψ values of 4.3° and 6.0° were observed, but this is still well within the thresholds defined in Table 2. The distribution of these two geometrical parameters is provided in Table 3 along with the same for other two systems. The angle φ is in the range of 79–90° and six angles around Cu are in the range of 90–135°. Like the D97 and D97p systems, in some cases one of the N(NO_2_^−^)-T2Cu-N(His) angle is larger and fell in the range of 140–157° (Table S1), hence, the overall structures are classified as tetrahedrally distorted trigonal structures.

2) Energetics of Cu(II) and Cu(I) states

In the Cu(II) case the individual energy differences between top-hat and N-bound structures are slightly higher than observed for the D97p systems; the maximum difference ranging up to 8 kcal/mol (see Table S1). The distribution of N-bound orientations in Cu(I) state slightly exceeds the top-hat orientations. In this system too, there is no clear correlation between the orientation of the NO_2_^−^ at the active site and the energy variation. The average ΔE for both Cu(I) and Cu(II) states is provided in Table 4. 

Here we see that for Cu(II) the average energy difference suggests that top-hat may be marginally favoured overall but with significant variation between snapshots as indicated by the large standard deviation. For Cu(I) the result is clearer cut with N-bound favoured over top-hat overall.

## 3. Discussion

The MD simulations in this work reveal an important consequence of the flexibility of the linker region in *Rp*NiR. They suggest that Tyr323 does not rigidly block the substrate access channel to the active site, rather it is flexible and can be displaced by the natural water dynamics in the resting state of the enzyme, causing the channel to open, which would allow NO or NO_2_^−^ to enter or leave the catalytic site. The MD corroborates the findings of Dong et al. for the D97N mutant [20], who observed displacement of Tyr323 for the NO-bound structure, resulting in an opening up of the channel. It is well known that sampling of the full configurational space is not possible in the time limit of typical all-atom MD simulations, and future work will use enhanced sampling methods to explore the flexibility of the Tyr323 residue and substrate entry to the T2Cu.

**Figure 4:**
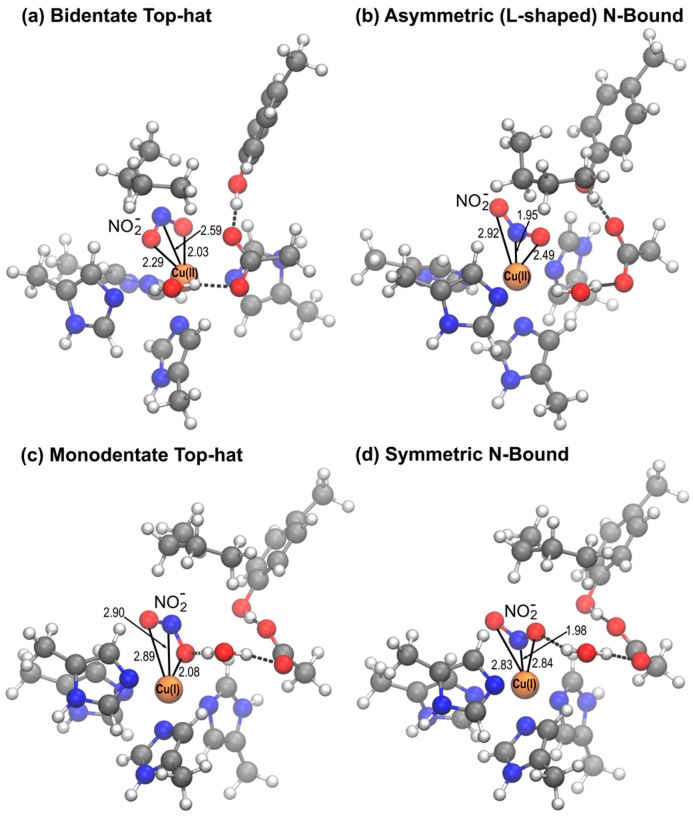
Representative structures of different orientations of NO_2_^−^ bound to T2Cu as classified by the geometric angles θ and ψ. (**a**) bidentate top-hat orientation, seen only in the Cu(II) state, (**b**) l-shaped N-bound orientation, more common in the Cu(II) state, (**c**) monodentate top-hat orientation observed in relaxed top-hat geometries after reduction to Cu(I), (**d**) symmetrical N-bound orientation observed in both the oxidised and reduced states on relaxation from the N-bound state.

A striking difference between the crystal structure of mutant *Rp*NiR and two-domain CuNiRs is the mode of NO_2_^−^ binding; in two-domain NiRs NO_2_^−^ binds in a bidentate top-hat orientation to T2Cu whereas in the *Rp*NiR mutant an N-bound orientation was observed [20]. In this work we have investigated the orientation of nitrite binding to T2Cu for both oxidized Cu(II) and reduced Cu(I) states of native and mutant *Rp*NiR. To categorize the different modes by which NO_2_^−^ can bind to the T2Cu we introduced two geometrical parameters, θ and ψ, and observed that the conformations obtained on QM/MM optimization of the nitrite bound structures clustered into particular ranges. Figure 4 shows representative structures of the four clusters of geometries as classified by θ and ψ, which we label bidentate top-hat, monodentate top-hat, l-shaped N-bound and symmetric N-bound.

There were however a few structures which adopted other orientations: one Cu(I) conformer in D97N optimized to a side-on NO_2_^−^ orientation, and one Cu(I) conformer in D97N and two Cu(I) conformers in D97p have θ values of approximately 150°, which lies in-between top-hat and side-on orientation. The side-on orientation was seen in MSOX data for the reduced Cu(I) state for *Ac*NiR [21], and hence could be a viable ligand orientation for binding to reduced *Rp*NiR.

The twist angle, φ, though originally used to define the type I Cu coordination geometry, can also be extrapolated to define the geometry around the T2Cu site [29]. In the case of *Rp*NiR, for all the systems studied φ is in the range of 80–90°, inclining towards trigonal structures. Considering the His-Cu-NO_2_^−^ and His-Cu-His angles along with φ, the structures are best classified as tetrahedrally distorted trigonal structures. In the work of Karllot et al. two distinct clustering of φ with maximum N(His)-Cu-N(His) angle was observed in the reported crystal structures of all two-domain NiRs [29]. One had φ in the range of 82–89° and maximum N(His)-Cu-N(His) angles around 111–125° whereas in the other φ was in the range of 65–77° and the maximum N(His)-Cu-N(His) angles around 134–162°. In our calculations on *Rp*NiR, however, we only find clustering of the former category. 

In the oxidized Cu(II) state the relative stabilities of the N-bound and top-hat states is not clear cut. However, for the reduced Cu(I) state there is a slight preference for the N-bound state in both the D97p and D97N systems and a clear preference for it in the D97 system (Table 4). The origin of the energy difference, however, cannot be solely related to the orientation of the NO_2_^−^ but to a complex interplay of the orientation as well as H-bond network in the active site. 

**Figure 5:**
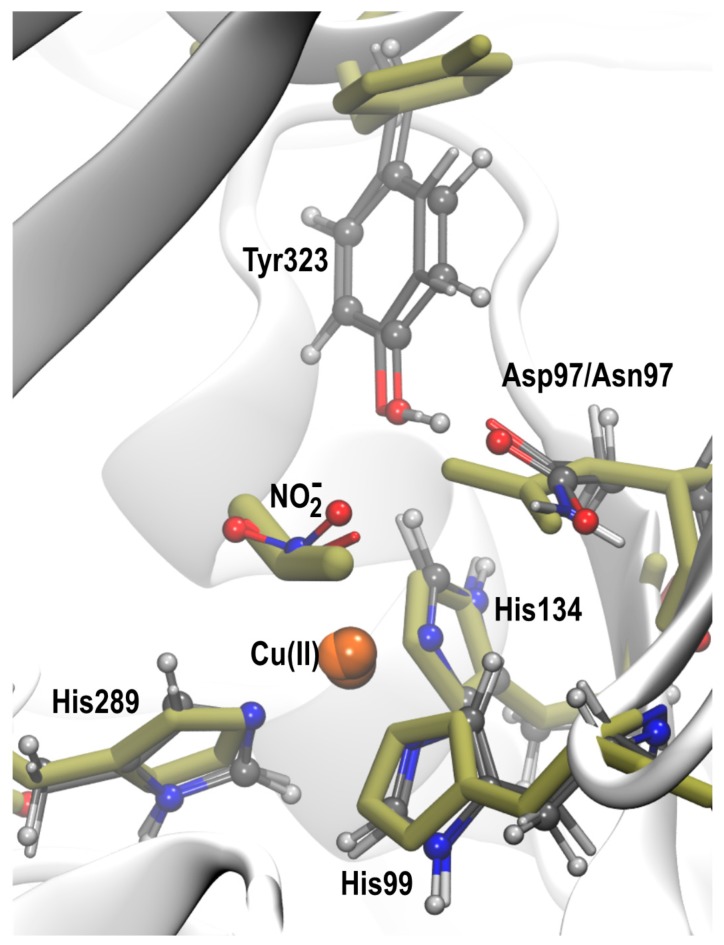
Superposition of the NO_2_^−^ bound X-ray structure of D97N *Rp*NiR (olive sticks) and two l-shaped N-bound conformers, obtained by simulations from an initially N-bound NO_2_^−^ to the Cu(II) state in the D97 and D97N systems.

Figure 5 shows the overlay of the X-ray structures of the nitrite-bound D97N mutant [20] and two simulated conformers, from the D97 and D97N systems, with similar l-shaped NO_2_^−^ orientations bound to Cu(II). In both systems, the Tyr323 was oriented in the substrate access channel as in the native *Rp*NiR crystal structure, rather than being displaced as observed experimentally for D97N. Apart from the difference in the Tyr323 position, the orientation of NO_2_^−^, the coordinated histidines and the other active site residues are all similar to the observed X-ray structure. In the crystal structure θ is 4.9° and ψ is 45.2° and the corresponding angles from the optimized structures are θ: 5.8°, 3.5° and ψ: 11.3°, 16.3°. Our findings indicate that the X-ray structure is consistent with the l-shaped N-bound NO_2_^−^ orientations observed for the Cu(II) state in *Rp*NiR and not the reduced Cu(I) state (SI, Figure S2), owing to the symmetrical N-bound orientation we observe in the reduced state. Such an l-shaped orientation is not primarily observed for two-domain CuNiRs in the Cu(II) state where a bidentate top-hat orientation of NO_2_^−^ is mostly prevalent.

## 4. Materials and Methods

Nitrite binding to native *Rp*NiR has not been structurally characterised experimentally to date and only very recently has nitrite binding in a mutant (D97N) been reported [20]. We used a native structure of *Rp*NiR in its resting state as a template to study the mode of nitrite binding to the T2Cu site. The native crystal structure at 1.01 Å resolution [9] was taken from the PDB databank (PDB ID: 3ZIY). The structure was first ‘cleaned’ for MD simulations by removing all double occupancy mainchain and sidechain atoms and partial water molecules, keeping those with greater fractional occupancy or lower B-factors. The homotrimeric biological unit was generated by symmetry operations using the clean version. Hydrogen atoms were added and the protonation states of the titratable residues apart from those at the active site were adjusted to be consistent with neutral pH using the propKa module of the PDB2PQR suite [33], followed by visual inspection of the local side-chain environments. The protonation states of the catalytically important residues, Asp97 (D97) and His240, were treated carefully. The residue His240 was singly protonated as HSD but the residue Asp97, the main residue involved in proton transfer, was considered in both its deprotonated and protonated forms in the native state. An in silico mutation to obtain D97N was performed on the native *Rp*NiR structure, so that Tyr323 retained the same initial orientation in the substrate channel/T2Cu pocket in all three systems being investigated: D97, D97p and D97N.

The prepared structures corresponding to the three systems were then solvated with a 15 Å layer of TIP3P water [34]. The electroneutrality of the D97, D97p and D97N systems were maintained by adding 6, 3, and 3 sodium counterions, respectively. Explicit all-atom MD simulations were performed on these systems using NAMD 2.9 [35] with the CHARMM36 force field [36]. These simulations employed Langevin dynamics with periodic boundary conditions at 293 K. Long range electrostatics were treated by the Particle Mesh Ewald method. In the NPT simulations the pressure was maintained with the Langevin piston method. Both the systems were initially subjected to 5000 steps of conjugate gradient (CG) minimization to eliminate any unphysical contacts. Next, the water and ions were equilibrated using an NVT ensemble, keeping the protein fixed for 1 ns. This was followed by 5000 steps of CG minimization and 5 ns equilibration under an NPT ensemble, keeping the backbone harmonically restrained (5 kcal^−1^mol^−1^ Å^2^) and the coordination spheres of both the T1 and T2 sites (Cu(His)_2_(Met)(Cys^−^) and Cu(His)_3_(H_2_O), respectively) constrained at their crystallographic coordinates. The simulation was continued for another 75 ns after removing the backbone restraints and allowing the coordinated water to move freely. The trajectories obtained from the MD simulations were analysed using VMD [37].

Snapshots were taken from the equilibrated MD trajectory as a starting point for QM/MM optimizations. The location of Tyr323 in the active site and the H-bond interaction of Tyr323 with Asp/Asn97 were used as the essential criteria for selecting the snapshots of both the native and mutant systems as detailed in the results section for each system. To obtain NO_2_^−^ bound structures, these selected snapshots were overlaid with the analogous “top-hat” NO_2_^−^ coordinates from the nitrite bound *Ac*NiR structure [38]. The water(s) coordinated to Cu and any nearby water molecules that were in unphysically close steric interaction with the overlaid NO_2_^−^ ion were then deleted. The nitrite coordinates were flipped to create analogous starting structures for the N-bound optimisations. In the absence of experimental data of the binding mode, this approach ensured that there was no bias in the initial starting structures towards either the top-hat or N-bound orientations.

The forces acting on the monomers along the simulation diverge, thus, the conformational arrangement at the catalytic sites which are housed at the interphase of two monomeric units are independent of one another, providing three independent starting structures in each snapshot. All these structures were subjected to QM/MM optimization of the nitrite bound T2Cu site following the standard QM/MM partition scheme. The QM region consists of the three histidine residues coordinating T2Cu (His99, His134 and His289), the NO_2_^−^ ligand, the three important active site residues Asp/Asn97, His240, and Ile242, the Tyr323 side chain, and any water molecules immediately H-bonded to Asp/Asn97, His240, Tyr323 and the NO_2_^−^ ligand. The residues were cut at the neutral Cα-Cβ bond and were capped with H-atoms to satisfy the valency for the QM calculations. Atoms within 7 Å of the QM region remained unconstrained during QM/MM geometry optimizations while the remaining atoms were frozen. The QM/MM optimizations were carried out for both the oxidized Cu(II) and reduced Cu(I) states of Cu starting from two orientations of NO_2_^−^: the η^2−^OO (bidentate) ‘top-hat’ and η^1^-N (monodentate) ‘N-bound’, to ensure that the potential energy surface was sufficiently explored. QM/MM calculations were performed with the Tcl-based version of the ChemShell computational chemistry environment [39,40], using ORCA [41] and DL_POLY [42] for the density functional theory and MM calculations respectively, and the DL-FIND module for geometry optimisations [43]. The electrostatic embedding scheme with charge shift correction was used to represent the surrounding MM partial charge distribution. The density functional B3LYP [44] with the DFT-D3 dispersion correction [45] was used for the QM atoms during geometry optimization and def2^−^SVP basis sets were used for all QM atoms except Cu, which was treated with the def2^−^TZVP basis set [46]. The CHARMM36 force field was used for the MM region.

## Figures and Tables

**Table 1:** Average distances between the T2Cu ion and the centres of mass of Asp/Asn97 and Tyr323 sidechains during 75 ns of unconstrained MD simulation. The equivalent crystal structure values are provided for comparison. Distances are in Å.

		D97	D97p	D97N	Crystal
Asp97/Asn97-Cu	Chain A	4.45 ± 0.18	4.61 ± 0.23	4.76 ± 0.32	4.48 (D97) [4.30] ^a^
	Chain B	4.46 ± 0.18	4.58 ± 0.24	4.60 ± 0.29	
	Chain C	4.57 ± 0.19	4.88 ± 0.23	4.96 ± 0.34	
Tyr323-Cu	Chain A	7.37 ± 0.19 9.83 ± 0.49 ^b^	7.38 ± 0.24	7.22 ± 0.22	7.16 [9.83] ^a^
	Chain B	7.33 ± 0.18	7.38 ± 0.23	7.35 ± 0.26	
	Chain C	7.35 ± 018	7.35 ± 0.20	7.21 ± 0.20	

**^a^** From crystal structure of NO_2_^−^ bound D97N (PDB ID: 5OBO). ^b^ Distance calculated following displacement of Tyr323 in chain A of D97 at ~69 ns in the MD trajectory.

**Table 2:** Angular thresholds for the characterisation of nitrite binding orientations.

Altitudinal Geometry	θ	Azimuthal Geometry	ψ
N-bound	0–20°	l-shaped	10–30°
		Symmetrical	0–10°
Top-Hat	160–180°	Bidentate	170–180°
		Monodentate	140–170°
Side-on	70–110°	n/a	

**Table 3:** Distribution of θ and ψ angles for the optimised conformations.

Geometrical Parameters and Distribution		Cu(II)			Cu(I)		
		D97	D97p	D97N	D97	D97p	D97N
Bidentate top-hat	θ/°	175.8 (1.1)	178.1 (1.6)	177.7 (1.7)	-	-	-
	ψ/°	174.2 (0.6)	175.0 (1.1)	174.1 (2.7)	-	-	-
	% structures ^b^	100	100	100	-	-	-
Monodentate top-hat	θ/°	-	-	-	175.5 (3.9)	174.5 (4.8)	175.3 (5.4)
	ψ/°	-	-	-	156.7 (2.4)	160.4 (5.6)	158.8 (5.3)
	% structures ^b^	-	-	-	83.3 ^a^	71.4 ^a^	66.7 ^a^
l-shaped N-bound	θ/°	5.8 (1.1)	6.1 (1.5)	5.1 (2.4)	-	-	-
	ψ/°	12.6 (1.5)	12.2 (1.5)	15.8 (1.0)	-	-	-
	% structures ^b^	66.7	71.4	33.3	-	-	-
Symmetrical N-bound	θ/°	5.4 ^c^	5.0 ^c^	5.8 (3.1)	5.8 (1.3)	7.1 (3.5)	4.3 (3.1)
	ψ/°	6.6 ^c^	4.9 ^c^	6.4 (1.9)	1.0 (0.7)	3.3 (1.9)	2.4 (1.8)
	% structures ^b^	33.3	28.6	66.7	100	100	100

^a^ Outliers are not reported in the table, see text and Table S1. ^b^ The percentage of structures which optimized to the orientations provided in the first column compared to the original starting structure. ^c^ Average over two structures only.

**Table 4:** Average energy difference ΔE = E_top-hat_ − E_N-bound_ and standard deviation in kcal/mol for NO_2_^−^ binding in D97, D97p and D97N.

System	Number of Starting Structures	Oxidation State	Average ΔE Energy in kcal/mol (std)
D97	6	Cu(II)	−0.27 (2.96)
		Cu(I)	8.66 (6.39)
D97p	7	Cu(II)	0.24 (2.73)
		Cu(I)	4.64 (7.66)
D97N	9	Cu(II)	−1.22 (4.29)
		Cu(I)	5.17 (4.87)

## Supplementary Materials

The supplementary materials are available online, Figure S1: Overlap of the NO_2_^−^ bound X-ray structure of D97N and the frame at 79.6 ns of D97 MD in its resting state. Chain A, where the Tyr is displaced (Figure 3 in main text) is highlighted here. The coordination around the T2Cu site and active site residues Asp97 and Tyr323 illustrate the similarity in the position of Asp97 and displacement of Tyr323 in MD that structurally matches the mutant crystal structure; Figure S2: Overlap of the NO_2_^−^ bound X-ray structure of D97N and two symmetrical N-bound conformers obtained from an initially N-bound NO_2_^−^ in the Cu(I) state of the D97 and D97N systems; Table S1: Geometric parameters and energies for the optimized conformers obtained from the D97, D97p and D97N systems. The distances are Cu-N, Cu-O1 and Cu-O2, respectively, where O1 and O2 are the two equivalent O-atoms of NO_2_^−^.
