# Supplementary material for: A QM/MM Study of Nitrite Binding Modes in a Three-Domain Heme-Cu Nitrite Reductase
Source: Molecules. 2018 Nov 16;23(11):2997. doi: 10.3390/molecules23112997 (PMC6278305; doi:10.3390/molecules23112997)
Supplement: Supplementary file 1 [file molecules-23-02997-s001.pdf]

molecules

Supplementary Material

A QM/MM Study of Nitrite Binding Modes in a Three-Domain Heme-Cu Nitrite Reductase

Kakali Sen[1, 2], Michael A. Hough[1], Richard W. Strange[1], Chin W. Yong[2] and Thomas W. Keal[2,*]

[1] School of Biological Sciences, University of Essex, Wivenhoe Park, Colchester, Essex, CO4 3SQ, United Kingdom; mahough@essex.ac.uk (M.A.H.); rstrange@essex.ac.uk (R.W.S.)
[2] Scientific Computing Department, STFC Daresbury Laboratory, Warrington, Cheshire, WA4 4AD, United Kingdom; kakali.sen@stfc.ac.uk (K.S.); chin.yong@stfc.ac.uk (C.W.Y.)
* Correspondence: thomas.keal@stfc.ac.uk (T.W.K.); Tel.: +44-1925603849

Contents

Figure S1: Overlap of the NO_2^- bound X-ray structure of D97N and the frame at 79.6 ns of D97 MD in its resting state. 2

Figure S2: Overlap of the NO_2^- bound X-ray structure of D97N and two symmetrical N-bound conformers obtained from an initially N-bound NO_2^- in the Cu(I) state of the D97 and D97N systems. 3

Table S1: Geometrical parameters and energies for the optimized conformers obtained from the D97, D97p and D97N systems. 4-10

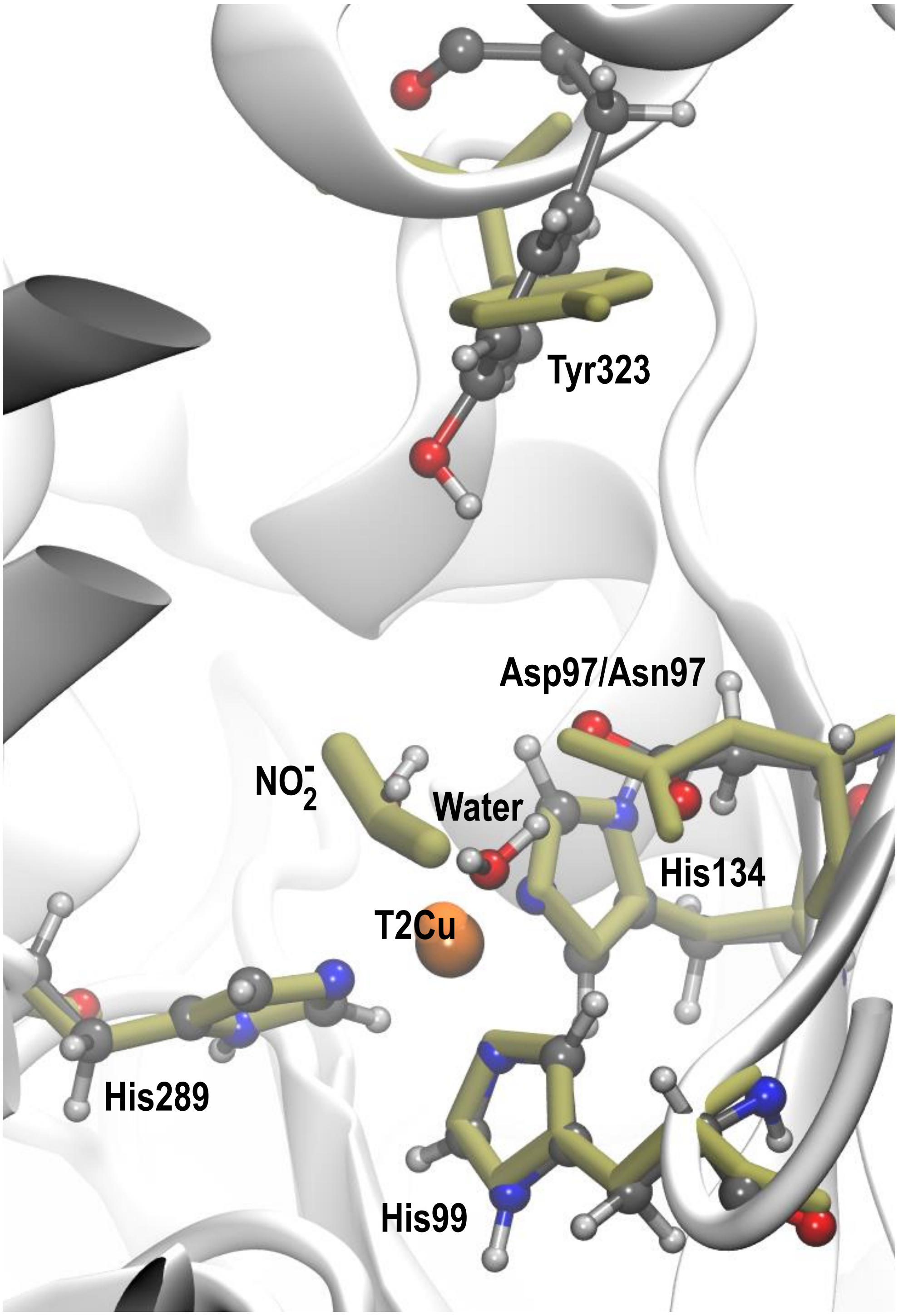

Figure S1. Overlap of the NO_2^- bound X-ray structure of D97N and the frame at 79.6 ns of D97 MD in its resting state. Chain A, where the Tyr is displaced (Figure 3 in main text) is highlighted here. The coordination around the T2Cu site and active site residues Asp97 and Tyr323 illustrate the similarity in the position of Asp97 and displacement of Tyr323 in MD that structurally matches the mutant crystal structure.

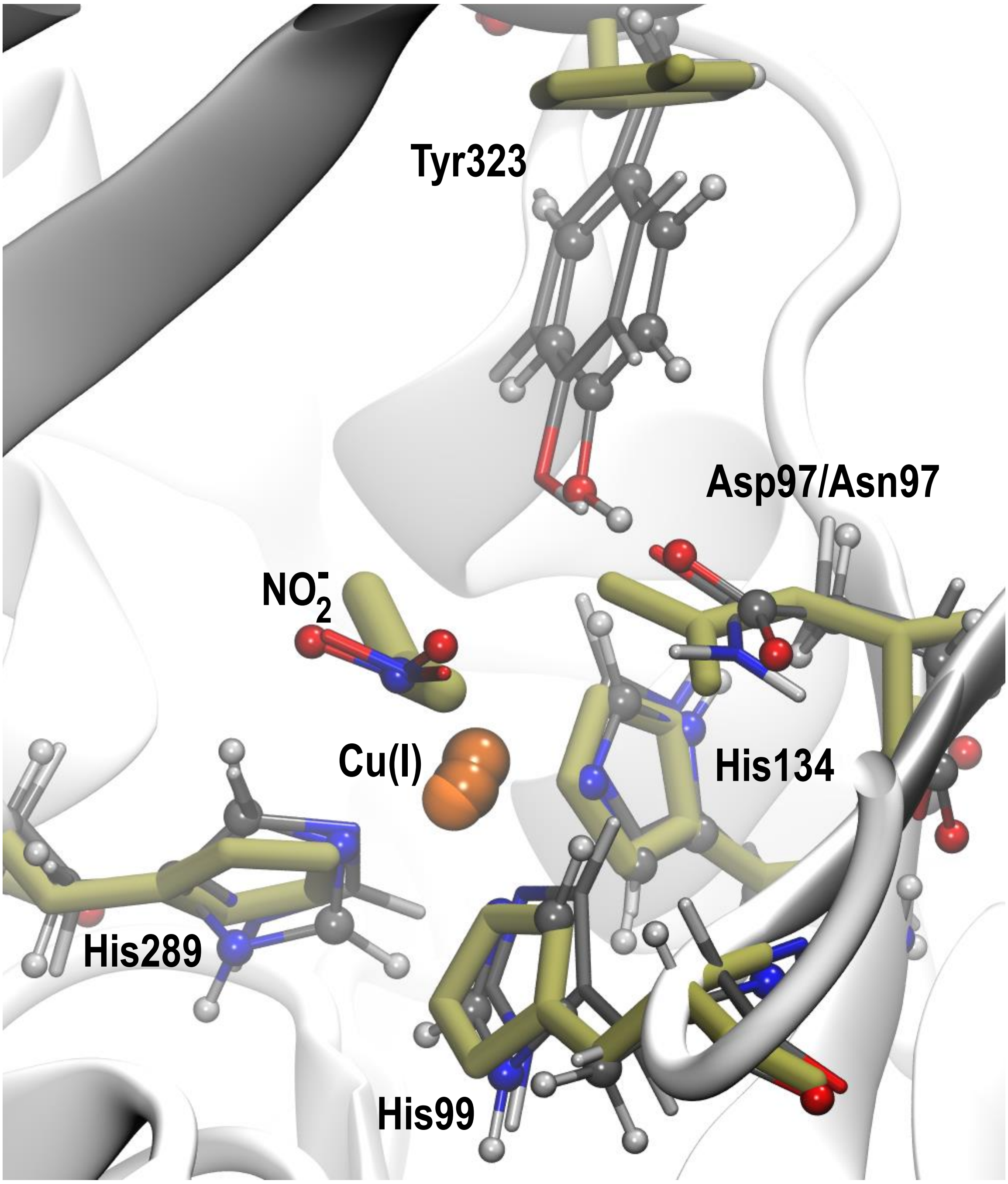

Figure S2. Overlap of the NO_2^- bound X-ray structure of D97N and two symmetrical N-bound conformers obtained from an initially N-bound NO_2^- in the Cu(I) state of the D97 and D97N systems.

Table S1. Geometric parameters and energies for the optimized conformers obtained from the D97, D97p and D97N systems. The distances given are Cu-N, Cu-O1 and Cu-O2, respectively, where O1 and O2 are the two equivalent O-atoms of NO_2^-.

**D97**		distances (Å)	NO_2^--Cu-His (°)	His-Cu-His (°)	Phi (ϕ°)	Theta (θ°)	Psi (ψ°)	Orientation	Energy (H)	ΔE (kcal/mol)
Conformer 1 Cu(II)	A: Top-hat	2.59, 2.03, 2.29	142.2, 112.8, 89.3	97.5, 121.5, 93.7	87.1	176.9	173.7	Bidentate Top-hat	-3871.865111	-5.54
	A: N-bound	1.95, 2.92, 2.49	142.1, 101.7, 98.4	98.7, 116.5, 100.6	88.4	4.9	14.6	L-shaped N-bound	-3871.856276	
	B: Top-hat	2.59, 2.03, 2.30	144.4, 112.2, 88.1	97.1, 121.2, 93.8	86.6	176.1	173.5	Bidentate top-hat	-3871.677285	0.96
	B: N-bound	1.99, 2.87, 2.67	162.2, 97.1, 84.1	98.8, 118.0, 95.1	84.3	8.9	6.9	Symmetrical N-bound	-3871.678814	
	C: Top-hat	2.58, 2.04, 2.26	142.9, 113.1, 89.8	96.7, 118.8, 94.8	86.4	176.6	174.6	Bidentate top-hat	-3871.713256	0.09
	C: N-bound	1.95, 2.89, 2.54	146.3, 102.4, 95.7	98.0, 111.9, 101.0	87.0	5.8	11.3	L-shaped N-bound	-3871.713393	
Conformer 1 Cu(I)	A: Top-hat	2.90, 2.89, 2.08	111.4, 120.9, 107.7	102.4, 110.5, 102.5	85.7	179.9	158.6	Monodentate top-hat	-3871.858416	5.78
	A: N-bound	1.98, 2.84, 2.83	113.2, 131.4, 103.2	101.4, 105.9, 96.1	85.2	6.4	0.4	Symmetrical N-bound	-3871.867633	
	B: Top-hat	2.87, 2.89, 2.01	147.5, 110.1, 84.2	102.3, 100.8, 87.3	89.4	177.5	158.0	Monodentate top-hat	-3871.665150	20.99
	B: N-bound	1.98, 2.81, 2.86	109.3, 137.0, 103.1	100.3, 103.4, 97.4	87.0	7.30	1.2	Symmetrical N-bound	-3871.698601	
	C: Top-hat	2.88, 2.90, 2.04	151.5, 106.7, 87.9	100.6, 105.7, 92.3	87.5	177.3	158.6	Monodentate top-hat	-3871.692928	5.85
	C: N-bound	2.00, 2.89, 2.80	114.1, 129.2, 103.0	100.0, 105.8, 101.4	84.5	6.1	1.6	Symmetrical N-bound	-3871.702252	

Conformer 2 Cu(II)	A: Top-hat	2.60, 2.03, 2.32	142.5, 117.2, 82.8	97.9, 115.7, 93.8	85.0	173.9	174.0	Bidentate top-hat	-4018.466208	3.49
	A: N-bound	1.98, 2.88, 2.67	159.4, 100.3, 86. 9	98.8, 107.6, 94.6	86.1	2.0	6.2	Symmetrical N-bound	-4018.471770	
	B: Top-hat	2.56, 2.02, 2.25	141.6, 115.5, 88.0	97.8, 115.5, 94.7	85.9	175.7	174.5	Bidentate top-hat	-3865.609923	-0.63
	B: N-bound	1.95, 2.89, 2.55	156.3, 99.3, 85.7	100.8, 113.4, 97.5	84.5	7.4	11.7	L-shaped N-bound	-3865.608915	
	C: Top-hat	2.58, 2.05, 2.25	144.2, 113.6, 88.3	96.4, 118.4, 94.3	86.3	175.7	175.1	Bidentate top-hat	-3865.735260	0.00
	C: N-bound	1.94, 2.90, 2.50	146.8, 101.8, 96.5	97.9, 111.0, 101.1	87.4	5.3	12.8	L-shaped N-bound	-3865.735259	
Conformer 2 Cu(I)	A: Top-hat	2.97, 3.11, 2.06	143.3, 113.9, 79.0	102.8, 104.1, 92.6	84.0	159.1	149.4	Monodentate top-hat	-4018.441324	6.87
	A: N-bound	1.97, 2.82, 2.84	117.7, 126.0, 102.0	103.9, 104.6, 98.3	85.2	3.6	2.1	Symmetrical N-bound	-4018.452279	
	B: Top-hat	2.90, 2.98, 2.00	145.2, 112.7, 81.6	102.1, 101.9, 90.7	86.3	170.8	153.8	Monodentate top-hat	-3865.570745	3.00
	B: N-bound	1.99, 2.85, 2.82	113.6, 132.0, 101.1	100.8, 106.0, 98.3	84.2	6.4	0.4	Symmetrical N-bound	-3865.575533	
	C: Top-hat	2.89, 2.97, 2.00	146.5, 113.1, 81.6	100.3, 103.4, 87.8	88.8	172.2	154.4	Monodentate top-hat	-3865.691786	9.43
	C: N-bound	1.98, 2.86, 2.80	112.9, 128.6, 104.0	101.1, 106.8, 99.9	85.1	5.2	0.5	Symmetrical N-bound	-3865.706807	

**D97p**		distances (Å)	NO_2^--Cu-His (°)	His-Cu-His (°)	Phi (ϕ°)	Theta (θ°)	Psi (ψ°)	Orientation	Energy (H)	ΔE (kcal/mol)
Conformer 1 Cu(II)	A: Top-hat	2.54, 2.24, 2.00	109.6, 109.8, 125.9	111.4, 101.4, 97.9	88.5	180.0	174.4	Bidentate top-hat	-3867.843640	0.40
	A: N-bound	1.96, 2.91, 2.56	139.6, 104.4, 96.8	100.0, 117.6, 99.7	87.7	5.7	12.6	L-shaped N-bound	-3867.844272	
	B: Top-hat	2.62, 2.02, 2.34	138.6, 104.7, 98.0	99.8, 125.0, 94.5	89.6	176.2	173.1	Bidentate top-hat	-3944.248645	-2.83
	B: N-bound	1.97, 2.92, 2.56	140.6, 105.2, 96.8	99.6, 117.6, 98.2	88.2	8.5	11.1	L-shaped N-bound	-3944.244141	
	C: Top-hat	2.53, 2.20, 2.02	114.9, 106.2 124.4	112.9, 100.3, 97.4	87.1	177.4	174.8	Bidentate top-hat	-4020.628102	2.52
	C: N-bound	1.96, 2.78, 2.74	142.7, 100.1, 96.1	102.8, 117.6, 98.8	89.6	2.9	0.2	Symmetrical N-bound	-4020.632119	
Conformer 1 Cu(I)	A: Top-hat	3.00 3.21 2.10	136.0, 101.6, 95.6	106.5, 112.0, 104.0	88.1	153.2	147.8	Between monodentate top-hat and side-on	-3867.911744	7.34
	A: N-bound	2.00, 2.89, 2.81	134.5, 111.2, 99.9	102.1, 108.7, 97.8	88.9	11.3	5.8	Symmetrical N-bound	-3867.923439	
	B: Top-hat	3.04, 3.15, 2.08	130.6, 119.7, 86.0	104.9, 113.6, 95.4	84.7	168.7	151.0	Monodentate top-hat	-3944.305058	-0.15
	B: N-bound	2.00, 2.78, 2.93	144.3, 110.2, 88.0	103.4, 112.2, 90.3	88.9	1.8	4.8	Symmetrical N-bound	-3944.304812	
	C: Top-hat	2.78, 2.09, 2.63	120.8, 101.7, 121.8	108.6, 103.4, 99.2	83.9	178.9	167.0	Monodentate top-hat	-4020.673116	8.74
	C: N-bound	1.97, 2.85, 2.82	126.6, 116.1, 103.2	105.3, 103.6, 98.0	89.9	3.9	1.7	Symmetrical N-bound	-4020.687044	
Conformer 2 Cu(II)	C: Top-hat	2.54, 2.21, 2.02	111.5, 109.3, 128.5	109.0, 100.2, 96.7	88.0	178.6	175.3	Bidentate top-hat	-3870.943614	-2.68
	C: N-bound	1.96, 2.90, 2.57	139.3, 104.6, 99.0	97.4, 118.2, 100.1	87.6	6.5	11.2	L-shaped N-bound	-3870.939341	

Conformer 2 Cu(I)	C: Top-hat	2.91, 2.07, 3.14	113.4, 95.9, 127.4	105.4, 109.5, 103.3	81.8	145.0	146.1	Between monodentate top-hat and side-on	-3871.001556	-2.28
	C: N-bound	1.96, 2.83, 2.79	129.1, 108.5, 109.6	104.2, 104.9, 98.0	87.1	9.5	2.3	Symmetrical N-bound	-3870.997926	
Conformer 3 Cu(II)	A: Top-hat	2.61, 2.07, 2.31	138.8, 103.4, 98.9	96.4, 133.0, 93.1	89.5	176.0	174.8	Bidentate top-hat	-3870.066464	-0.14
	A: N-bound	1.97, 2.96, 2.53	143.5, 100.7, 98.5	98.1, 123.7, 96.6	89.9	5.2	14.7	L-shaped N-bound	-3870.066239	
	B: Top-hat	2.52, 2.19, 2.00	118.4, 102.8, 127.4	104.8, 100.8, 99.5	86.1	179.0	175.9	Bidentate top-hat	-3870.018931	4.83
	B: N-bound	1.97, 2.90, 2.58	143.5, 101.1, 97.4	97.6, 117.4, 101.3	87.8	4.7	11.4	L-shaped N-bound	-3870.026631	
	C: Top-hat	2.51, 2.15, 2.02	118.9, 105.4, 128.1	102.1, 102.3, 96.4	84.5	179.5	176.4	Bidentate top-hat	-3870.058196	-0.39
	C: N-bound	1.95, 2.88, 2.59	142.0, 104.0, 98.4	97.1, 119.2, 98.4	88.2	7.1	9.6	Symmetrical N-bound	-3870.057578	
Conformer 3 Cu(I)	A: Top-hat	2.92, 2.90, 2.08	136.6, 104.6, 96.0	105.2, 115.9, 98.5	89.1	170.3	159.9	Monodentate top-hat	-3870.124830	-3.44
	A: N-bound	1.99, 2.87, 2.80	133.1, 106.5, 105.9	103.9, 110.2, 95.9	86.9	9.5	3.6	Symmetrical N-bound	-3870.119353	
	B: Top-hat	2.89, 2.90, 2.04	123.1, 108.3, 113.0	98.9, 106.9, 104.9	88.5	179.0	157.8	Monodentate top-hat	-3870.068921	18.45
	B: N-bound	2.00, 2.87, 2.84	133.9, 110.3, 104.3	100.7, 103.8, 100.3	88.7	8.7	4.2	Symmetrical N-bound	-3870.098319	
	C: Top-hat	2.88, 2.79, 2.12,	116.5, 117.3, 105.8	97.8, 117.5, 101.1	78.8	175.5	163.4	Monodentate top-hat	-3870.123358	3.83
	C: N-bound	1.98, 2.82, 2.86	121.4, 126.0, 100.4	98.2, 111.1, 95.8	78.4	4.8	0.4	Symmetrical N-bound	-3870.129466	

**D97N**		distances (Å)	NO_2^--Cu-His (°)	His-Cu-His (°)	Phi (θ°)	Theta (θ°)	Psi (ψ°)	Orientation	Energy (H)	ΔE (kcal/mol)
Conformer 1 Cu(II)	A: Top-hat	2.62, 2.05, 2.36	139.8, 97.4, 106.1	99.3, 122.8, 94.9	86.4	178.4	173.2	Bidentate top-hat	-3847.527062	-0.74
	A: N-bound	1.96, 2.91, 2.61	149.0, 94.6, 99.2	102.9, 111.6, 97.8	87.0	2.7	8.5	Symmetrical N-Bound	-3847.525883	
	C: Top-hat	2.63, 1.99, 2.40	133.9 103.0, 111.0	99.8, 112.9, 95.8	86.7	177.6	171.2	Bidentate top-hat	-3923.895746	7.67
	C: N-bound	1.96, 2.84, 2.68	137.3, 105.2, 99.3	102.6, 113.3, 98.5	89.7	7.4	3.8	Symmetrical N-bound	-3923.907967	
Conformer 1 Cu(I)	A: Top-hat	3.04, 3.14, 2.15	145.8, 98.1, 94.0	105.4, 110.1, 100.5	89.3	177.9	152.6	Monodentate top-hat	-3847.578835	6.68
	A: N-bound	2.03, 2.93, 2.86	143.8, 99.6, 105.8	102.5, 104.6, 96.0	85.8	8.7	0.0	Symmetrical N-bound	-3847.589481	
	C: Top-hat	2.97, 3.13, 2.12	118.0, 121.6, 89.1	112.3, 108.3, 101.6	79.4	149.4	148.2	Between monodentate top-hat and side-on	-3923.960995	-0.92
	C: N-bound	1.98, 2.83, 2.87	124.0, 122.9, 103.1	103.2, 101.5, 96.3	89.5	1.5	1.8	Symmetrical N-bound	-3923.959530	
Conformer 2 Cu(II)	A: Top-hat	2.60, 2.03, 2.32	142.7, 110.5, 89.9	93.8, 132.1, 94.4	86.6	176.6	173.0	Bidentate top-hat	-3924.744903	-6.69
	A: N-bound	1.95, 2.94, 2.47	137.6, 111.3, 94.7	100.4, 112.7, 98.3	86.5	7.90	16.4	L-shaped N-bound	-3924.734243	
	B: Top-hat	2.54, 2.08, 2.13	125.4, 106.8, 119.2	104.4, 103.2, 95.3	83.2	179.4	179.9	Bidentate top-hat	-3924.723438	0.36
	B: N-bound	1.96, 2.85, 2.66	136.3, 106.8, 100.3	103.2, 111.6, 97.3	86.6	8.0	5.4	Symmetrical N-bound	-3924.724008	
	C: Top-hat	2.57, 2.02, 2.25	128.5, 105.4, 116.0	99.6, 109.6, 96.0	86.5	179.0	175.7	Bidentate top-hat	-3924.766377	-0.73
	C: N-bound	1.96, 2.90, 2.60	141.4, 103.9, 96.9	101.4, 113.1, 99.4	88.5	7.1	8.7	Symmetrical N-bound	-3924.765207	

Conformer 2 Cu(I)	A: Top-hat	2.67, 2.11, 3.45	106.4, 104.7, 125.4	104.3, 113.6, 100.3	85.1	88.1	87.8	Side-on	-3924.790435	7.28
	A: N-bound	1.99, 2.85, 2.85	119.4, 121.7, 106.2	102.4, 105.9, 98.1	84.9	2.0	1.7	Symmetrical N-bound	-3924.802035	
	B: Top-hat	2.83, 2.14, 2.65	115.0, 111.4, 111.0	108.6, 111.9, 98.4	85.8	179.2	168.1	Monodentate top-hat	-3924.763224	11.42
	B: N-bound	1.97, 2.84, 2.84	118.6, 124.9, 104.3	104.6, 102.5, 96.8	87.1	3.4	0.8	Symmetrical N-bound	-3924.781426	
	C: Top-hat	2.84, 2.23, 2.57	105.0, 114.5, 115.4	103.1, 111.5, 105.8	88.8	179.8	172.1	Bidentate top-hat	-3924.820505	4.81
	C: N-bound	1.99, 2.83, 2.88	122.6, 124.1, 100.0	103.2, 104.1, 97.5	83.6	4.2	2.0	Symmetrical N-bound	-3924.828169	
Conformer 3 Cu(II)	A: Top-hat	2.53, 2.21, 1.99	107.5, 112.4, 127.8	107.9, 101.8, 97.5	89.1	179.2	174.9	Bidentate top-hat	-3847.425518	-4.09
	A: N-bound	1.96, 2.95, 2.51	142.4, 103.4, 98.3	98.8, 118.4, 97.4	89.2	4.0	14.6	L-shaped N-bound	-3847.418994	
	C: Top-hat	2.58, 2.00, 2.31	139.1, 116.4, 88.8	96.7, 118.4, 96.0	85.1	176.0	172.2	Bidentate top-hat	-3923.653382	-5.53
	C: N-bound	1.95, 2.93, 2.47	136.7, 102.1, 100.6	98.8, 117.0, 102.9	88.3	3.5	16.3	L-shaped N-bound	-3923.644575	
Conformer 3 Cu(I)	A: Top-hat	2.89, 2.05, 2.96	119.9, 99.4 121.2	106.3, 109.9, 99.4	79.9	164.7	154.7	Monodentate top-hat	-3847.457441	6.51
	A: N-bound	2.01, 2.96, 2.78	115.4, 117.4, 117.3	99.7, 107.8, 95.9	86.5	0.6	4.3	Symmetrical N-bound	-3847.467811	
	C: Top-hat	2.87 2.83 2.09	106.4, 122.0, 113.2	102.2, 105.2, 106.3	89.4	174.4	159.6	Monodentate top-hat	-3923.706236	11.61
	C: N-bound	1.98 2.81 2.85	103.3, 139.6, 106.6	100.7, 101.6, 98.0	89.4	9.5	2.3	Symmetrical N-bound	-3923.724743	
Conformer 4 Cu(II)	A: Top-hat	2.58 2.04 2.27	129.4, 103.0, 108.0	97.3, 125.6, 96.1	88.6	178.6	174.8	Bidentate top-hat	-3923.753399	-2.59
	A: N-bound	2.12 2.87 2.99	96.4, 108.1, 141.6	94.6, 108.3, 92.4	88.0	1.1	5.9	Symmetrical N-bound, penta-coordinated	-3923.749277	

								with water		
	C: Top-hat	2.62 2.04 2.38	143.4, 109.4, 87.3	96.9, 133.8, 92.6	87.2	174.4	171.7	Bidentate top-hat	-3923.756654	1.41
	C: N-bound	1.97 2.85 2.67	157.2, 98.9, 86.3	99.4, 124.3, 94.6	85.9	8.7	6.0	Symmetrical N-bound	-3923.758896	
Conformer 4 Cu(I)	A: Top-hat	2.91 2.07 2.91	101.1, 114.8, 120.4	102.4, 113.8, 100.1	89.7	177.9	158.4	Monodentate top-hat	-3923.806413	-0.60
	A: N-bound	2.01 2.86 2.88	110.0, 111.8, 128.6	100.4, 104.4, 97.5	87.3	5.5	2.4	Symmetrical N-bound	-3923.805462	
	C: Top-hat	2.92 2.90 2.07	137.8, 110.4, 90.2	103.7, 115.9, 96.4	86.4	177.9	158.9	Monodentate top-hat	-3923.812761	-0.29
	C: N-bound	2.01, 2.74, 2.96	139.3, 110.8, 93.0	103.7, 110.8, 94.4	88.6	3.1	6.0	Symmetrical N-bound	-3923.812297	
